# Spatiotemporal Evolution of the Water System’s Structure and Its Relationship with Urban System Based on Fractal Dimension: A Case Study of the Huaihe River Basin, China

**DOI:** 10.3390/e27010092

**Published:** 2025-01-20

**Authors:** Hailong Yu, Bin Yu, Xiangmin Zhang, Yong Fan, Sai Wen, Shanshan Jiao

**Affiliations:** 1 School of Tourism and Planning, Pingdingshan University, Pingdingshan 467000, China; hlyu0912@163.com (H.Y.); ws15100345881@163.com (S.W.); 2 College of Urban and Environmental Sciences, Central China Normal University, Wuhan 430079, China; 3 School of Geographical Sciences, Xinyang Normal University, Xinyang 464000, China; xyzxm@126.com; 4 School of Artifcial Intelligence, Shenzhen Polytechnic University, Shenzhen 518055, China; gisfanyong@163.com

**Keywords:** water system structure, urban system structure, fractal dimension, spatiotemporal evolution, relationship characteristic, the Huaihe River Basin

## Abstract

The formation and development of cities are inseparable from a certain scale of water resources. The information contained in the morphological structures of cities and water systems is often overlooked. Exploring the spatiotemporal evolution of water system structures (WSS) and urban system structures (USS) can reveal the “urban–water” relationship from a new perspective. The Huaihe River Basin (HRB) was selected as the case area, based on the theory of fractal dimensions, grid dimension and multifractal spectrum methods were used to depict the structural evolutionary characteristics of water systems and urban systems from different dimensions. Then, through a comparative analysis of fractal parameters and spectral lines, the characteristics and changing patterns of the “urban-water” relationship in the HRB from 1980 to 2019 were revealed. The results indicate the following: (1) The water system structure in the HRB is complex and exhibits distinct scale characteristics, showing improvement overall and at larger scales while continuously degrading at smaller scales. (2) Both the water system and urban system exhibit increasingly complex spatial development characteristics; however, the USS continues to optimize over time, while the WSS experiences degradation. (3) The development patterns of the water system and urban system are significant differences in the HRB. Urban development mainly relies on outward expansion, whereas the water system is primarily characterized by intensive enhancement. (4) Because of the rapid development of urban areas, water scarcity may occur in densely populated urban areas or larger cities in the future. The research results can serve as a scientific reference for urban planning and water resource management in the HRB.

## 1. Introduction

Water has a dedicated sustainable development goal (SDG 6) in the United Nations’ 2030 Agenda for Sustainable Development, alongside Zero Hunger (SDG 2) and Climate Action (SDG 13) [1], aimed at providing clean drinking water and sanitation for all and ensuring sustainable management of these resources. Water resources are the most important environmental elements for sustaining human survival and development [2]. Water systems are carriers and manifestations of water resources, and they support these resources, upon which humans rely for survival, as well as underpin the sustainable development of regional economies and societies [3]. The frequent and complex changes in water systems have profound and long-lasting impacts on both the natural and cultural environments of watersheds. Since ancient times, the origins, prosperity, and decline of cities have been closely related to the changes in and evolution of water systems. The evolution of water systems directly affects the supply conditions of regional water resources, leading to regional water resource issues [4]. With the acceleration of urbanization, the impact of human activities on water systems has intensified, leading to a reduction in the number of water systems and a decline in connectivity [5], which trigger a series of problems, such as weakened flood regulation capacity, declining water quality, and a deteriorating ecological environment [6,7].

The changes in watershed water systems, variations in water resources, and their impacts on urban development are currently hot topics in hydrology and water resource research. In September 2019, the Chinese government identified the coordinated promotion of ecological environment protection and high-quality development in the Yellow River Basin as a major regional development strategy. In October of the same year, it was proposed that “we must adhere to the principle of determining cities, populations, and industrial development based on water, treating water resources as the most rigid constraint, and reasonably planning population, urban, and industrial development” [8]. Watershed water systems and urban systems are important carriers of the natural environment and human activities [9]. Within a specific watershed, a corresponding urban system typically develops, and the two interact and influence each other throughout their historical development. Clarifying the relationship characteristics between water system evolution and urban development is key to adhering to the principle of “determining by water” and collaboratively promoting watershed ecological protection and high-quality urban development.

The impacts of urbanization on changes in water systems and water resource security has become a focal point of national and regional sustainable development [10,11]. In the study of water system evolution, river network density, water surface ratio, fractal dimension, and river curvature are typically used to characterize the quantity, structure, and morphological features of water systems, thereby exploring the temporal and spatial evolutionary characteristics of regional water systems and their response mechanisms [12,13]. Research on water system evolution in historical periods is often based on historical literature or historical atlases, primarily focusing on long-term macro analysis. Current research is largely based on topographic maps and interpretation of data from remote sensing images, depicting more detailed characteristics of water system evolution [14]. The combined effects of climate change and human activities are the main reasons for changes in watershed water resources [15,16]. Water systems influence the spatial form and distribution patterns of cities, with areas of higher urbanization levels experiencing more drastic changes in water systems [17]. The development of an urban system complicates the evolution of water systems, and the spatiotemporal evolutionary relationship between watershed water systems and urban systems remains to be further explored.

The urban system plays key roles in issues such as social, economic, and environmental sustainability [18]. Although cities occupy only 2% of the world’s total land area, they contribute 70% of the world’s gross domestic product, and more than half of the world’s population lives in cities [19]. Scientific understandings of the spatial pattern characteristics and evolution laws of regional urban systems are of great significance for the rational planning of regional urban layouts and optimization of urban systems [20]. In the early 20th century, German geographer W. Christaller established Central Place Theory and opened the door for research on urban systems [21], and theories and methods related to urban systems have been extensively explored. Modern mathematical methods, such as entropy theory, fractal theory, cellular automata, and machine learning, are widely applied in the study of urban systems [22,23,24,25], and there have been attempts to propose a theoretical framework for urban systems and their evolution [26]. Research on urban systems exhibits the typical characteristics of data diversification, method integration, and scale refinement [27].

Because of the heterogeneity of geographical environments and the selectivity of human activities, certain water resources are always associated with specific human activities. Human activities that ignore the carrying capacity of water resources and the excessive concentration of human activities that deviate from the carrying capacity of water resources have become deep-seated reasons for the imbalance in the human–water relationship and intensification of human–water conflicts in contemporary times. Scientifically understanding the relationship between humans and water, solving water-related problems with socio-economic development, and achieving a harmonious coexistence between humans and water in regions have become important topics of universal concern among countries around the world [28]. Currently, indicator-based methods are commonly used to comprehensively evaluate regional human–water relationships [29], including proposed indexes such as the Water Stress Index [30], Water Vulnerability Index [31], Water Accessibility Index [32], Water Poverty Index [33], Water Resource Carrying Capacity Index [34], and Water–Human Harmony Index [35]. However, a unified evaluation index and method system has not yet been established [36]. Watershed water systems and urban systems are important carriers of water resources and human activities, respectively; yet there are few reports on the study of their relationship. In the face of complex human–water relationship issues, new methods and models need to be further explored.

Fractal dimension is another measure of entropy, which is closely related in dynamical systems [37]. Fractal theory was first proposed by Mandelbrot [38], and it can generally be divided into simple fractals and multifractals. Although simple fractals can generally describe the complexity of a research object, they cannot reflect local features; multifractal methods can reveal more spatial information within a system [39,40]. The establishment of fractal geometry has provided geographers with effective mathematical tools to describe the spatial forms of geographical elements; it is widely applied in numerous fields such as rivers, landforms, and towns [41]; and it has been used to compare and analyze the fractal structural characteristics of geographic elements on different scales [42]. Existing research indicates that both urban systems and water systems exhibit significant fractal characteristics [43,44], and there is a correlation and greater similarity between watershed water systems and urban systems [45]. The development of fractal correlation theories and methods has provided a new method and idea for exploring the structural changes and relationships between water systems and urban systems.

With the increasing pressure from global water scarcity, it is estimated that by 2025 1.8 billion people will live in countries or regions with water shortages, and two-thirds of the world population’s demand for clean water will not be met [46]. China is recognized as a water-scarce country, with a population accounting for about 20% of the world’s total, but its freshwater resources only account for about 5–7% of the global total, resulting in a serious problem related to water resource shortages [47]. The Huaihe River Basin (HRB) is located in the geographical transition zone between northern and southern China, and the Huaihe River system has experienced significant changes in its history due to the comprehensive influence of external environmental changes, resulting in the failure to form a complete urban system in the basin for a long period. The HRB is densely populated and has a high intensity of human activities, with the per capita water resources in the basin being only one-fifth of the national average, indicating significant water resource issues. It is urgently needed to analyze the relationship between the evolution of watershed water systems and development of urban systems, explore ways to optimize water resource management and urban systems, and promote harmonious water–human relationships and regional sustainable development.

The formation and development of any city is inseparable from the support of a certain scale of water resources, and cities have a strong spatial dependence on watershed water systems. Urban development has profoundly influenced and changed the quantity and structure of watershed water systems through various forms of water resource development and utilization. There exists a close and complex relationship between the water system and urban system in basins, but the relationship characteristics and mechanisms have not been clearly elucidated. As research on regional human–water relationships, the scientific questions in this study are as follows: (1) What are the spatial structural characteristics of a water system and urban system in a complete natural space (the HRB)? How have they evolved over time? (2) What are the structural characteristics of relationships between water systems and urban systems, and how can they be quantitatively explained and described? In this paper, the HRB was selected as the research area, and based on multi-source remote sensing data and land use datasets/platform, we extracted water system and urban construction land data for different periods between 1980 and 2019. Then, the network dimension method and multifractal spectrum method were used to characterize the structural evolution of the water system and urban system from different dimensions. Finally, through a comparison and analysis of the fractal parameters, fractal spectrum lines, and their changes, the characteristics and changing patterns of the “urban-water” relationship in the HRB from 1980 to 2019 were revealed. This can provide a theoretical framework and methodological reference for the promotion of watershed water resource management, optimization of urban systems, and implementation of a “water-based urban planning” strategy.

## 2. Study Region

**Figure 1:**
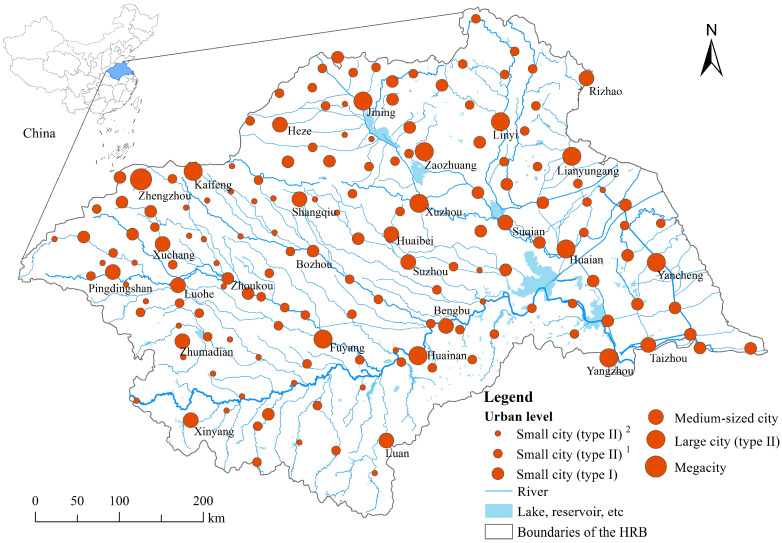
Location of the Huaihe River Basin. The water systems and urban settlements data came from the National Geographic Information Resource Directory Service System (http://www.ngcc.cn/, accessed on 20 April 2021). The classification of urban levels refers to the “Notice on Adjusting the Standards for Classifying Urban Sizes” issued by China. The difference is that small cities (type II) are divided into the following two levels, represented by superscript format of numbers 1 and 2: small cities (type II) ^1^ with populations of 100,000–200,000 and small cities (type II) ^2^ with populations fewer than 100,000.

The Huaihe River Basin (the HRB) is located in the central eastern part of China, starting from the Tongbai Mountains and Funiu Mountains in the west, and bordering the Yellow Sea in the east, situated between the Yangtze River and Yellow River basins (111°55′–121°20′ E, 30°55′–36°20′ N), with a total area of approximately 270,000 km^2^. It is one of the seven major river basins in China (Figure 1). The HRB is situated in the transitional zone of the northern and southern climates in China, where the evolution of the water system has been frequent and complex throughout history, with frequent occurrences of flooding- and drought-related meteorological disasters. The interannual and seasonal variations in precipitation in the watershed are significant, and their spatial distribution is uneven, with a multi-year average precipitation of 878 mm, increasing from northwest to southeast. The average annual total of water resources in the basin is 812 × 108 km^3^, of which surface water resources account for approximately 75%, representing about 2.9% of the national total. The HRB covers the following five provinces—Henan, Anhui, Shandong, Jiangsu, and Hubei—including 40 cities, and it plays an important role in China’s socio-economic development pattern. In 2018, the permanent population of the basin was approximately 164 million, with an average population density of 607 people/km^2^, which is 4.2 times the national average population density. The GDP of the basin was CNY 8.36 trillion, accounting for approximately 9.26% of the national-level total GDP (data are from The Huaihe River Commission of the Ministry of Water Resources, China). With the rapid development of the economy and society, the contradiction between the supply of and demand for water resources in the HRB has become increasingly prominent, placing it among the severely water-scarce basins in China. This basin, with less than 3% of the country’s total water resources, supports approximately 11.8% of the population and 11.7% of the arable land, produces one-sixth of the country’s grain, and contributes 9.26% of the national GDP. Relevant studies have indicated that the pressure on water resources in the HRB is 4–5 times the national average, ranking first among the seven major basins in environmental pressure, which is an important factor restricting the healthy development of economy and society in the basin [48]. Exploring the structural evolution of the water system and its relationship with the urban system can provide references for water resource management, urban system optimization, and regional high-quality development in the new era.

## 3. Methods and Models

**Figure 2:**
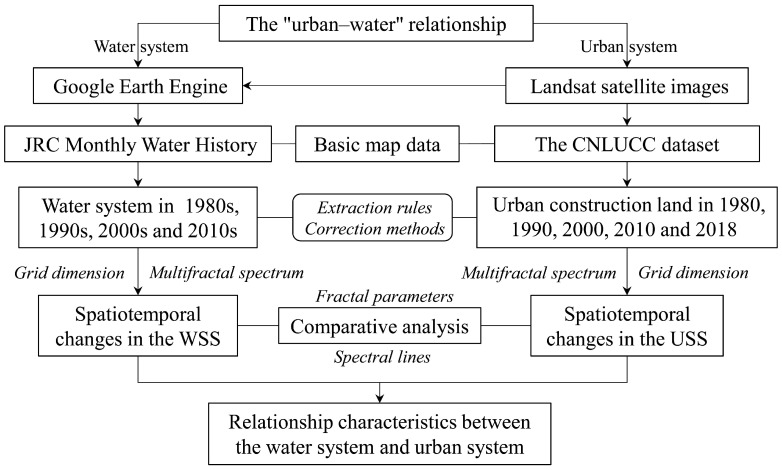
Flowchart of the research design. The CNLUCC dataset refers to the “China Multiperiod Land Use Remote Sensing Monitoring Dataset”. The WSS and USS refer to water system structures and urban system structures, respectively.

The spatial distribution patterns of geographical elements generally exhibit irregularities and a featureless scale, such as the distribution of urban systems and water systems, which are difficult to accurately quantify using traditional geometric methods and mathematical modeling based on characteristic scales [49]. The establishment of fractal geometry provided geographers with an effective mathematical tool to describe the spatial forms of geographical elements. Fractal dimension is a characteristic parameter of fractals, which can be used to summarize complex geographic spatial data into a simple number and reveal the underlying spatiotemporal information of geographical elements [50]. Due to the complexity and variability of geographical elements such as water systems and cities, in reality, different fractal elements have different characteristics. Simple fractals can only reflect their basic states and cannot comprehensively reveal spatial features at different scales. Therefore, two fractal methods, the grid dimension method and multifractal spectrum, are used to describe the spatial morphological characteristics of the water system and urban system. A flowchart of the research design is shown in Figure 2.

### 3.1. Grid Dimension Method

**Figure 3:**
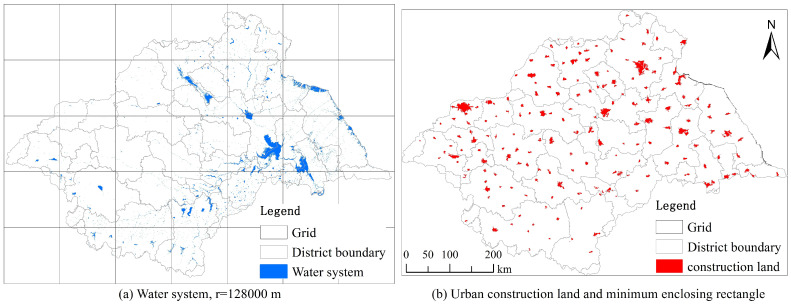
Grid division map of the water system and urban construction land in the Huaihe River Basin.

The grid dimension method is a single-fractal method that measures the scale invariance within a study area by dividing it into uniform grids. It can examine the spatial filling degree and balance of geographical objects within a study area. This fractal method is easy to understand, computationally simple, and widely applicable, but the spatial information it can reveal is limited [22,49,51]. During the calculation, the study area is first divided into grids with a side length of *r*, and then by continuously changing the size of *r*, the number of grids, *N_r_*, occupied by the study object is calculated. If the morphology of the research object conforms to fractal characteristics, then *r* and *N_r_* satisfy the following model [52]:

(1) $$N_{r}\propto r^{-D},$$

where *D* is the grid dimension. The specific calculation steps are as follows:

Firstly, using the Create Fishnet tool in ArcGIS 10.5 software, a series of grids of different sizes are created, with the grid side lengths, r, set to the following 9 levels: 500 m, 1000 m, 2000 m, 4000 m, …, 128,000 m. Figure 3a shows a map of the grid with the maximum side length level.

Secondly, using the Zonal Statistics as Table tool, with the grids as the area feature data and the water system and urban construction land as the value raster data, the number of occupied grids, *N_r_*, are counted.

Finally, according to Formula (1), the least squares method is used to perform linear fitting on ln*Nr* and ln*r*, where the negative slope represents the grid dimension, *D*.

### 3.2. Multifractal Models

Multifractals, also known as multi-scaling fractals, refer to fractals that possess two or more scaling processes, indicating that different regions, levels, or scales exhibit varying distribution probabilities or growth probabilities. Multifractals can acquire spatial information across different regions or scales, thereby revealing structural differences within complex systems [53]. The multifractal model is relatively complex compared to the grid dimension method, but it can reflect the fractal characteristics of areas with different densities, revealing more spatial information within the system.

The multifractal model consists of the following two sets of parameters: global and local. Global parameters focus on the whole, whereby all regions are within the measurement range during the calculation, including the generalized dimension, *D_q_*, and the *q*-order mass index, *τ*(*q*). Local parameters focus on specific areas and only measure regions of a certain density, reflecting multi-scale fractal characteristics on a microscopic scale, including the singularity index, *α*(*q*), and local dimension, *f*(*α*). Global and local parameters can be linked through the Legendre transform [54].

A. For global parameters, the generalized correlation dimension, *D_q_*, and mass index, *τ*(*q*), based on Rényi entropy are expressed as follows [55]:

(2) $$D_{q}=-\underset{r\to 0}{\lim}\frac{I_{q}(r)}{\ln r}=\left\{\begin{array}{c} \frac{1}{q-1}\underset{r\to 0}{\lim}\frac{\ln\sum_{i=1}^{N(r)}P_{i}{\left(r\right)}^{q}}{\ln r},(q\neq 1)\\ \underset{r\to 0}{\lim}\frac{\sum_{i=1}^{N(r)}P_{i}(r)\ln P_{i}(r)}{\ln r},(q=1) \end{array}\right.,$$

(3) $$\tau (q)=(q-1)D_{q},$$

where *I*(*r*) is the Rényi entropy; *i* and *r* are the sequence number and scale of the fractal element, respectively; *N*(*r*) is the number of non-empty fractal elements at *r*-scale; *P_i_* is the distribution probability or growth probability of the *i*-th fractal element, and the larger is *P_i_*, the higher the density of the area; and *q* represents the moment, with a larger value of *q* reflecting information about regions with a higher density (higher growth probability) and vice versa. When *q* = 0, 1, or 2, this represents the three basic parameters of multifractals, namely, the capacity dimension, *D*_0_; information dimension, *D*_1_; and correlation dimension, *D*_2_. By changing the moment, *q*, the fractal characteristics of different density regions can be reflected [45]. The generalized dimension, *D_q_*, and the mass index, *τ*(*q*), have a certain equivalent correspondence, so the generalized dimension spectrum (*q*-*D_q_* spectrum) is usually considered for use in global parameter analyses.

B. For local parameters, the singularity index, *α*(*q*), and its corresponding local dimension, *f*(*α*),s are defined as follows:

(4) $$\alpha (q)=\underset{r\to 0}{\lim}\frac{1}{\ln r}\sum_{i=1}^{N_{\left(r\right)}}\mu_{i}\ln P_{i},$$

(5) $$f(\alpha )=\underset{r\to 0}{\lim}\frac{1}{\ln r}\sum_{i=1}^{N_{\left(r\right)}}\mu_{i}\ln\mu_{i}$$

where *μ_i_* is the probability reconstruction based on the growth probability, *P_i_*. According to *P_i_*, *μ_i_*, and Equations (4) and (5), the singularity index and local fractal dimension can be estimated using the least squares method.

For the *i*-th fractal element, the calculation method for its growth probability, *P_i_*, is as follows:

(6) $$P_{i}=\frac{A_{i}}{A}=\frac{A_{i}}{\sum_{i=1}^{N(r)}A_{i}},$$

where *A_i_* is the area of geographic features (water system or urban construction land) in the *i*-th grid, and *A* is the total area.

The “*μ*-weight method” is used to estimate the singularity index, *α*(*q*), and the local dimension, *f*(*α*), making it convenient for calculations and easy to control errors [56], the calculation formula for the reconstruction probability, *μ_i_*, is as follows:

(7) $$\mu_{i}=\frac{P_{i}^{q}}{\sum_{i=1}^{N(r)}P_{i}^{q}}$$

where *μ_i_* can be calculated according to different *P_i_* and *q* values.

The specific calculation steps are as follows:

Firstly, taking the study area (the HRB) as the boundary and its minimum enclosing rectangle as the maximum box (Figure 3b), by dividing the maximum box into four, four into sixteen, etc., 10 rectangular grids with different scales are created.

Secondly, the area of the geographic features (water system or urban construction land) within the grid is determined, and, according to Formulas (6) and (7), the growth probability, *P_i_*, and reconstruction probability, *μ_i_*, are calculated based on different moments, *q*.

Finally, based on *P_i_*, *μ_i_*, and Formulas (2) to (5), the least squares method is used to estimate the global and local parameters, respectively. The generalized dimension spectrum (*q*-*D_q_* spectrum) and the local fractal spectrum (*α*-f(*α*) spectrum) are drawn. The local fractal spectrum is also referred to as the singular spectrum.

The multifractal spectrum is typically analyzed using the *q*-*D_q_* spectrum line and the *α*-*f*(*α*) spectrum line [57]. In the *q*-*D_q_* spectrum, the spectrum line is an inverted S-shaped, monotonically decreasing squeezed curve. As q approaches positive and negative infinity, the values of *D_q_* reach their respective lower and upper limits, representing the fractal elements with the minimum and maximum growth probabilities. The *α*-*f*(*α*) spectrum line can reveal more detailed spatial structural characteristics of the research object. In the *α*-*f*(*α*) spectrum, *α* = *α*(*q*) represents a fractal element of a specific density, and the larger its value, the lower the corresponding set density. *f*(*α*) is the fractal element dimension with a specific density, where a larger value indicates a higher degree of spatial filling and uniformity of the fractal element at that density.

In a specific analysis, it is common to combine *α*(*q*) with *f*(*α*(*q*)) to form the local fractal spectrum (*α*-*f*(*α*) spectrum). When analyzing the *α*-*f*(*α*) spectrum, the peak values of the *α*-*f*(*α*) spectrum line, width of the singularity index Δ*α* (Δ*α* = α-∞ − α+∞), and height difference Δ*f* (Δ*f* = *f*(*α* + ∞)− *f*(*α* − ∞)) when the *α* − *f*(*α*) spectrum converges are commonly used interpretative parameters [58,59]. The explanations for each parameter are as follows [45]:

- Peak value of the α-f(α) spectral line: Since *α*-*f*(*α*) is a unimodal curve, the *f*(*α*) value ranges from 0 to *D*_0_, with *q* = 0 (*α*(0), *f*(*α*(0))) being its peak value. The larger the *α*(0) value, the higher the spatial filling degree of the research object; the larger the *f*(*α*(0)) value, the more complex the spatial structure.
- Δ*α* (*α*-∞–*α*+∞): The ratio of Δ*α* to the embedding space dimension 2 can define a spatial filling index. The larger the value, the greater the difference between the highest and lowest densities of the fractal object, the higher the spatial filling degree, and the stronger the heterogeneity. The Δ*α* was calculated using *α* − 40–*α* + 40 in this paper.
- Δ*f* (*f*(*α*+∞)–*f*(*α*-∞)): This is used to determine the fractal growth mode of the research object. When Δ*f* < 0, the *α-f*(*α*) spectral shape is unimodal and right-skewed (higher on the right, lower on the left), indicating that the fractal growth of the research object is primarily centered, representing an intrinsic enhancement mode. When Δ*f* > 0, the *α*-*f*(*α*) spectral shape is unimodal and left-skewed (higher on the left, lower on the right), indicating that the fractal growth of the research object is primarily outwardly diffusive, representing an extrinsic expansion mode. When Δ*f* ≈ 0, the *α*-*f*(*α*) spectrum shows a left–right symmetric arc, with the growth patterns of the highest and lowest density areas being similar. The Δ*f* is calculated using *f*(*α* + 40)–*f*(*α* − 40) in this paper.

## 4. Data Collection and Processing

### 4.1. Data Sources

In this study, the data types include water system data, urban system data, and basic map data.

(1) Water system data

The water system data came from the water thematic dataset of the JRC Monthly Water History (v1.2). This is based on the Google Earth Engine platform, sourced from 4,185,439 Landsat satellite images acquired between 16 March 1984, and 31 December 2019. The expert system classification method was applied to separately classify each raster into water and non-water, generating monthly water data from March 1984 to December 2019 with a spatial resolution of 30 m.

To objectively analyze the evolutionary characteristics of the watershed system, stable water bodies such as rivers and lakes should be taken as research objects, and some areas with temporary water accumulation, caused by low-lying or extreme precipitation, should be excluded. Therefore, we define areas covered by water for more than half of the year as stable water bodies—thus, a grid that judges water bodies as those that exist for at least 7 months each year—which can be considered as the real water body data. According to this rule, annual water body data for the HRB were generated for 1985 to 2019.

(2) Urban system data

The urban system data were mainly sourced from the China Multiperiod Land Use Remote Sensing Monitoring Dataset (CNLUCC), The CNLUCC dataset was established with the support of several national major scientific projects, covering land areas for the entire country in a multi-temporal land use database. The remote sensing image from the United States Landsat satellite is the main information source, and the classification results were obtained through human–computer interactive visual interpretation methods. Currently, a total of 10 periods of data from 1980 to 2020 have been released, with the land use types including six primary categories—arable land, forest land, grassland, water bodies, construction land, and unused land—as well as 25 secondary categories. The average classification accuracy reaches 75–85%, with a spatial resolution of 30 m. The data were downloaded for free from the Resource and Environment Data Cloud Platform (http://www.resdc.cn/Default.aspx, accessed on 11 November 2021).

To reduce errors caused by a single data source and interpretation method, artificial surfaces from the GlobeLand 30 dataset (National Geographic Information Resource Directory Service System http://www.webmap.cn/mapDataAction.do?method=globalLandCover, accessed on 21 April 2021) were referenced to correct the urban area data.

(3) Basic map data

The basic map data mainly included an administrative map and the boundaries of the HRB, which were taken from the National Geographic Information Resource Directory Service System (http://www.ngcc.cn/, accessed on 20 April 2021), with a scale of 1:1,000,000, and the Huaihe River Commission of the Ministry of Water Resources, P.R.C. (http://www.hrc.gov.cn/, accessed on 21 April 2021).

The differences in image resolution and quality should be preprocessed through missing strip repair, geometric correction, mosaic and cropping, projection conversion, resampling, etc. The remote sensing data and map data were all unified into an Albers conic projection (central longitude of 116° E and double standard parallels of 32° N and 35° N, respectively), and the 2000 National Geodetic Coordinate System (CGCS2000) was the frame of reference.

### 4.2. Calculation and Analysis

In this study, the steps in the calculation and analysis are as follows:

Firstly, the water system and urban construction land data are extracted.

**Figure 4:**
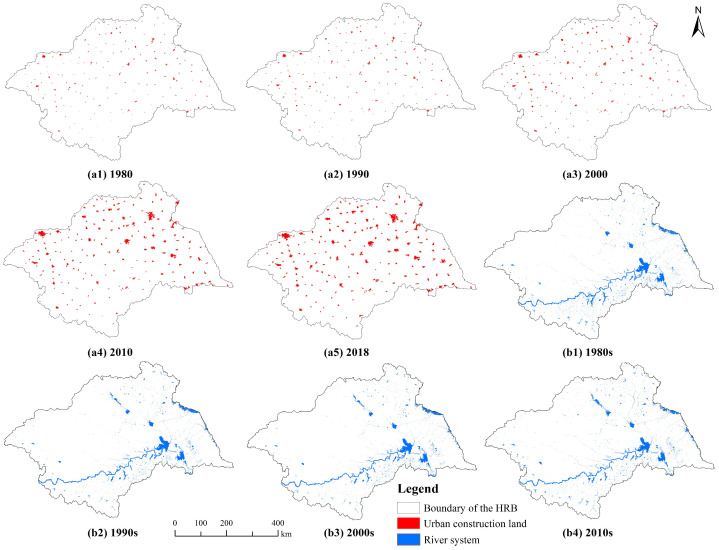
Spatial distribution maps of surface water bodies and urban construction land in the Huaihe River Basin from 1985 to 2019: (**a1**–**a5**) urban built-up areas in 1980, 1990, 2000, 2010, and 2018, respectively; (**b1**–**b4**) water systems in the 1980s, 1990s, 2000s, and 2010s, respectively.

Using Landsat remote sensing images from 1980 to 2018 as data sources, and referencing various land use classification datasets and construction land interpretation methods, urban construction land in the HRB for the years 1980, 1990, 2000, 2010, and 2018 was extracted (Figure 4(a1–a5)).

In order to accurately determine the spatiotemporal changes in water bodies, we divided the water bodies from 1985 to 2019 into four time periods—1985–1989, 1990–1999, 2000–2009, and 2010–2019—representing the water bodies of the 1980s, 1990s, 2000s, and 2010s, respectively. The extraction rule was as follows: stable water bodies during each time period represent the actual water bodies. Raster units that are classified as water bodies in half or more of the years within each time period are determined to be stable water bodies for that period, specifically. According to this rule, the water system data of the HRB in the 1980s, 1990s, 2000s, and 2010s were extracted (Figure 4(b1–b4)).

Secondly, the fractal dimension is calculated and the fractal spectrum is drawn.

According to Formula (1) and its calculation steps, the grid dimension calculation results for the water system over 4 periods and the urban system over 5 periods in the HRB are obtained, and the fitting graphs of the fractal logarithmic relationship are drawn. On this basis, further calculations and drawings of the multifractal spectrum and related parameters for the water system and urban system in the HRB are conducted according to Formulas (2) to (7).

Thirdly, the structural relationship between water system and urban system is analyzed.

Based on the above calculation results, the similarities and differences in the grid dimensions, fractal spectra, and related parameters are compared and analyzed between the water system and urban system, along with their trends in change, and the structural relationship characteristics between them are explored based on the connotations of the values and spectral lines.

## 5. Results

### 5.1. Characteristics of the Water System Structure in the HRB

#### 5.1.1. Grid Dimension Analysis of the Water System

**Figure 5:**
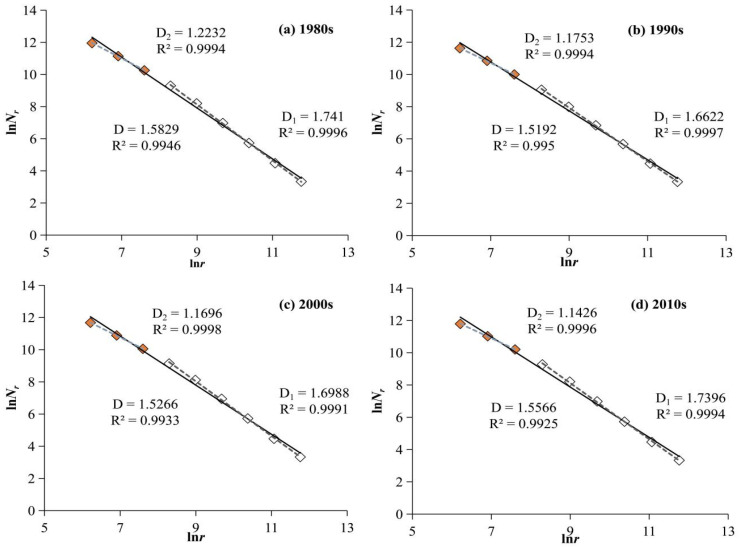
Fitting diagram of fractal logarithmic relationship of the water system in the Huaihe River Basin from 1980 to 2019: (**a**–**d**) refer to the 1980s, 1990s, 2000s, and 2010s, respectively. The solid line represents the whole, and *D* is the overall grid dimension. The dashed line represents segments, where *D*_1_ and *D*_2_ are the first-order scale zone grid dimension (the last 6 points, white squares) and the second-order scale zone grid dimension (the first 3 points, red squares).

The calculation results of the grid dimensions of the water system in the HRB for four periods and the fitting graphs of the fractal logarithmic relationships are shown in Figure 5 and Table 1.

In the real world, there are no standard fractals; fractal characteristics only exist within a certain scale range, which is referred to as the scale region [57]. As shown in Figure 4, the fitting curves of the grid dimensions of the water system in the HRB since the 1980s have two scale regions with different slopes, presenting a clear dual-scale structure. This indicates that the water system of the HRB exhibits fractal characteristics within the following two scale ranges (i.e., scale regions): 500–2000 m (3 points) and 4000–128,000 m (6 points). Further analysis of the grid dimension values and related parameters reveals the following fractal characteristics of the spatial distribution structure of water system:

First, overall, the fractal development of the water system in the HRB is insufficient, and the spatial structure shows a trend of degeneration. On the one hand, the fitting effect of the overall grid dimension, *D*, is poor. Studies have indicated that if the R^2^ value of the grid dimension fitting is greater than 0.996, it can be determined that the morphology is fractal [43]. The R^2^ value of the overall grid dimensions in the four periods of the HRB is less than 0.996, indicating insufficient development of the fractal structure of the spatial morphology of the entire basin’s water system. On the other hand, the self-radioactivity of the water system structure is continuously strengthening. The dual-scale structural characteristics of the water system are becoming increasingly evident. The differences in grid dimensions (*D*_1_–*D*_2_) between the scale areas in the 1980s, 1990s, 2000s, and 2010s was 0.5178, 0.4869, 0.5292, and 0.597, respectively, with the differences gradually increasing. This indicates that the self-radioactivity of the water system in the HRB is gradually strengthening, and its spatial structure is continuously degenerating.

Secondly, from 1980 to 2019, the grid dimension value shows an overall decreasing trend. Because of the poor quality or absence of remote sensing images in the 1980s, issues may arise in the interpretation of the water system, leading to discrepancies between the grid dimensions of the water system during this period and the overall trend. Therefore, data from this period are excluded from the analysis. From 1990 to 2019, the overall grid dimensions of the water system, *D*, and the grid dimension of the first-order scale zone, *D*_1_, show an increasing trend, while the grid dimension of the second-order scale zone, *D*_2_, exhibits a decreasing trend. This indicates the complexity of spatial changes in the water system structure of the HRB, with a noticeable increase in the spatial filling extent at larger scales. The overall grid dimension, *D*, and the first-order scale zone grid dimension, *D*_1_, for the four periods are 1.5829, 1.5192, 1.5266, and 1.5566 and 1.741, 1.6622, 1.6988, and 1.7396, respectively. After excluding the data for the 1980s, the increasing trend in the grid dimensions is very evident. The variation characteristics of the second-order scale zone grid dimension, *D*_2_, are different from *D* and *D*_1_. Regardless of whether the data for the 1980s are excluded, *D*_2_ shows a gradually decreasing trend. It can be seen that, from 1990 to 2019, the spatial filling extent of the HRB water system had different variation characteristics for different scale ranges, showing a significant increasing trend overall and at larger scales, while presenting a decreasing trend at smaller scales. This result indicates that the distribution of the water system in the HRB significantly improved over the past 30 years in general and on a larger scale, but there are still ongoing degeneration issues at smaller scales.

#### 5.1.2. Multifractal Spectrum Analysis of the Water System

**Figure 6:**
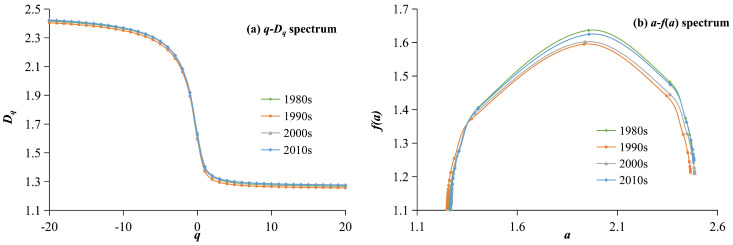
The *q*-*D_q_* spectrum and *a*-*f*(*a*) spectrum of the water system in the Huaihe River Basin from 1980 to 2019.

The *q*-*D_q_* spectrum and the *a*-*f*(*a*) spectrum of the water system in the HRB is shown in Figure 6. Because of quality-related issues in the interpretation of the water system in the 1980s, data from this period were not analyzed. From the generalized dimension, *q*-*D_q_*, spectrum of the water system (Figure 6a) and its parameters (Table 2), the following can be seen: Firstly, the overall differences in the *q*-*D_q_* spectrum of the water system in the HRB during various periods from 1990 to 2019 are relatively small, and as time progresses, the spectral lines show an upward trend. This indicates that the water system structure in the HRB has not changed significantly over the past 30 years, showing a slight expansionary trend, particularly evident between 1990 and 2000. Secondly, the differences in the translation speeds of the multifractal spectral lines in the regions with large and small *q* values are relatively small, indicating that the changes in the water system structure of the HRB are consistent in both dense and sparse areas of the water system. The generalized dimension reflects the spatial filling degree of geographical objects, and the smaller *q* value reflects information from low-density areas. The differentiation of curves across different periods is not significant, whether in the low-density area with *q* < −4 or in the high-density area with *q* > 3. This indicates that the structural changes in the water system in the HRB from 1980 to 2019 were relatively small, and the regional variations in the different densities of the water systems were generally consistent. Thirdly, the capacity dimension (*D*_0_), information dimension (*D*_1_), and relational dimension (*D*_2_) of the water system in the HRB all show an upward trend (Table 2). The higher the fractal dimension value, the greater the filling degree of the water system, indicating a more developed water system. Among them, the ratio of the information dimension to the capacity dimension, *D*_1_/*D*_0_, shows an increasing trend, indicating that the morphological differences in the water system in the HRB are decreasing.

The global parameters reflect the overall situation of the HRB’s water system, while local parameters focus on the parts and details. From the singularity spectrum of the water system’s *a*-*f*(*a*) curve (Figure 6b) and related parameters (Table 2), the following can be seen: Firstly, the spatial filling degree of the HRB water system has continuously increased, and the spatial structure has become increasingly complex from 1990 to 2019. The values of *α*(0) and *f*(*α*(0)) have gradually increased, from 1.9333 and 1.595 in the 1980s to 1.9588 and 1.6246 in the 2010s, respectively. The peak of the singular spectrum showed a trend of gradually increasing and shifting to the right, with the most significant changes from 2000 to 2019. Secondly, the widths of the singularity index, Δ*α*, for the three periods from 1990 to 2019 are 1.2169, 1.2171, and 1.2172, showing a gradually increasing trend, indicating that the differences between high-density and low-density areas of the HRB water system increased, with an increase in spatial filling degree and enhancement of the heterogeneity, leading to improved development of the water system. Third, the spectral lines all exhibit a single peak slightly skewed to the right, indicating that the spatial distribution of the HRB’s water system is primarily characterized by spatial aggregation, and the aggregation characteristics exhibit an increasing trend. The Δ*f* values for the three periods from 1990 to 2019 are −0.1461, −0.1308, and −0.1958, which are all less than 0 and exhibit a decreasing trend. It is evident that the spatial distribution of the HRB’s water system exhibits a significant aggregation pattern, with the development of the water system primarily characterized by internal improvement, which becomes more prominent over time.

### 5.2. Characteristics of the Urban System Structure in the HRB

#### 5.2.1. Grid Dimension Analysis of the Urban System

**Figure 7:**
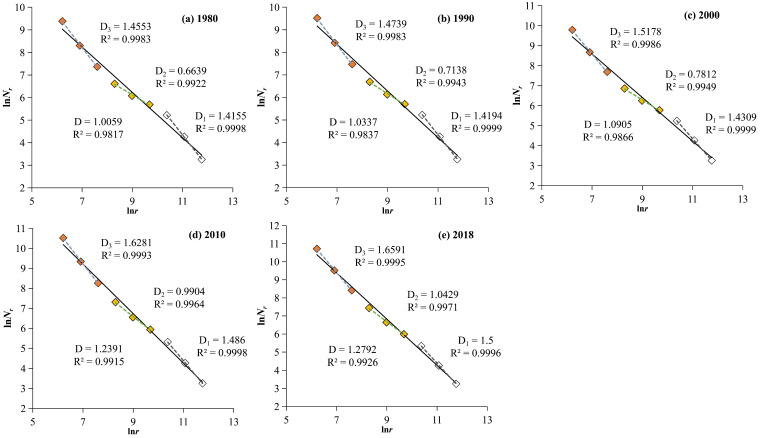
Fitting diagram of the fractal logarithmic relationship of the urban system in the HRB from 1980 to 2018. The solid line represents the whole, with *D* representing the overall grid dimension. The dashed line represents segments, where *D*_1_, *D*_2_, and *D*_3_ correspond to the first-order scale grid dimension (the last 3 points, white squares), the second-order scale grid dimension (the middle 3 points, yellow squares), and the third-order scale grid dimension (the first 3 points, red squares), respectively.

The calculation results of the grid dimensions of the urban system in the HRB for five periods and the fitting graph of the fractal logarithmic relationship are shown in Figure 7 and Table 3.

As shown in Figure 7, unlike the dual-scale fractal structure of the water system, the fitting curves of the urban grid dimensions in the HRB from 1980 to 2018 exhibit a three-scale structure with different slopes, indicating a strong self-affine fractal property of the urban areas in the basin. Fractal characteristics are observed in the following three scale ranges (i.e., scale zones): 500–2000 m (3 points), 4000–16,000 m (3 points), and 8000–128,000 m (3 points). Further analysis of the grid dimension values and related parameters (Table 3) reveals the following fractal characteristics of the spatial distribution structure of the urban areas in the HRB:

Firstly, the overall fractal development of the urban construction land in the HRB is insufficient, but its spatial structure shows a trend of optimization. The fitting effect of the overall grid dimension, *D*, for the five periods is poor, with the R^2^ values all less than 0.996. This indicates that the fractal structure of the urban spatial morphology in the HRB is underdeveloped, exhibiting fractal characteristics only within a relatively small scale range. From the difference in grid dimensions across various scale regions, it can be observed that from 1980 to 2018, the differences in grid dimensions *D*_3_–*D*_2_ and *D*_1_–*D*_2_, as well as the average difference, gradually decreased. This suggests that urban construction land in the HRB is evolving from a multifractal structure to a single-fractal structure, and urban areas are evolving from self-affine fractals to self-similar fractals. This reveals that the structure of the urban construction land in the HRB is continuously optimizing over time.

Secondly, the spatial filling degree of the urban areas in the HRB has been continuously increasing, and the urban structure has shown a clear trend of optimization at different scales. From 1980 to 2018, the overall grid dimension, *D*, and the grid dimensions *D*_1_, *D*_2_, and *D*_3_ for the three sub-scale regions gradually increased, from 1.0059, 1.4155, 0.6639, and 1.4553 in 1980 to 1.2792, 1.5, 1.0429, and 1.6591 in 2018, respectively. Additionally, R^2^ also showed a gradual increasing trend. This change was most evident during the period from 2000 to 2010, with the largest increase in all grid dimensions. This indicates that the fractal characteristics of the urban areas in the HRB have gradually become prominent over the past 40 years, with a significant improvement in spatial filling at both the overall and various scales, reflecting a continuous optimization trend in urban morphological spatial development.

#### 5.2.2. Multifractal Spectrum Analysis of the Urban System

**Figure 8:**
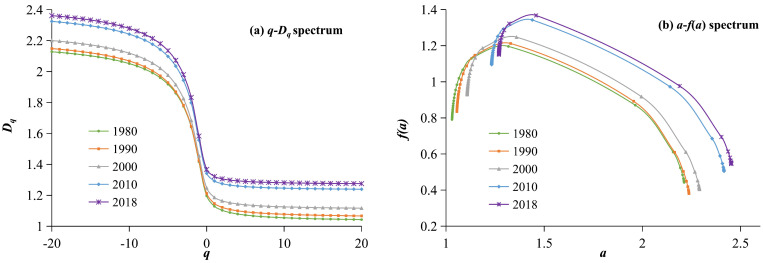
The *q*-*D_q_* spectrum and *a*-*f*(*a*) spectrum of the urban system in the Huaihe River Basin from 1980 to 2018.

The *q*-*D_q_* spectrum and *a*-*f*(*a*) spectrum of the urban system in the HRB is shown in Figure 8. From the generalized dimension, *q–D_q_*, spectrum of the urban construction land (Figure 8a) and its parameters (Table 4), the following can be observed: Firstly, from 1980 to 2018, the multifractal *q*-*D_q_* spectral lines exhibited significant differentiation across five periods, and gradually shifting upward with time, with the most significant shift occurring from 2000 to 2010. This indicates that the structure of the urban construction land in the HRB has undergone significant changes over the past 40 years, showing a clear expansion trend, with the most intense urban spatial expansion occurring from 2000 to 2010. Secondly, the translation speed of the multifractal *q–D_q_* spectrum lines varies across different ranges of q values. When *q* > 0, convergence occurs faster than when *q* < 0, indicating that the development of dense urban areas is not synchronized with that of sparse areas, and the fractal structure of dense areas is more developed. In the multifractal spectrum, *q* > 0 represents information from high-density areas, revealing the changing characteristics of large urban centers; *q* < 0 represents the information from low-density areas, revealing the changing characteristics of suburban areas of large cities and small- to medium-sized towns. When *q* < −3 and q > 1, the curves of each period show significant divergence, and the curves for 2010 and 2018 are both positioned at a high level, indicating that the development of various types of towns in the basin is relatively rapid, with the fastest growth occurring between 2000 and 2010. When *q* > 1, the curve rapidly converges to a nearly flat state, indicating that, compared to sparse urban areas or small- and medium-sized towns, the spatial structure of dense urban areas or large cities is more reasonably and orderly developed. Thirdly, from 1980 to 2018, the capacity dimension (*D*_0_), information dimension (*D*_1_), and relational dimension (*D*_2_) of the urban construction land in the HRB all exhibit a significant upward trend (Table 4), with the three fractal dimension values increasing from 1.1962, 1.1292, 1.1033 in 1980 to 1.3676, 1.3219, 1.3066 in 2018, respectively. This indicates that the filling degree of the urban construction land is continuously increasing, and the spatial structure of the urban areas is becoming more and more developed. At the same time, the ratio of the information dimension to the capacity dimension, *D*_1_/*D*_0_, also shows an increasing trend, indicating that the differences in the urban construction land patterns in the HRB are gradually decreasing.

Based on the singularity spectrum *a*-*f*(*a*) curve of the urban construction land (Figure 8b) and related parameters (Table 4), the details of the development of the urban spatial structure in the HRB can be observed. The main characteristics are as follows: First, from 1980 to 2018, the spatial filling degree of the urban construction land in the HRB continuously increased, and the spatial structure became increasingly complex. The values of *α*(0) and *f*(*α*(0)) gradually increased, rising from 1.3178 and 1.1962 in 1980 to 1.4587 and 1.3676 in 2018, respectively. The peak values of the spectral lines exhibit a trend of gradually increasing and shifting to the right, with the most significant changes occurring from 2000 to 2010. Secondly, the width Δ*α* of the singularity index for the five periods from 1980 to 2018 shows a trend of gradually increasing over time, indicating that the differences in the maximum and minimum densities of the urban construction land in the HRB have increased, with an increase in the spatial filling degree and enhancement of the heterogeneity, leading to a continuous acceleration of the urban expansion. Thirdly, the spectral lines all exhibit a clear single-peak left skew, indicating that the spatial form of towns in the HRB is primarily characterized by a dispersed distribution, and the trend of outward expansion is continuously strengthening. The Δ*f* values for the five periods of 1980, 1990, 2000, 2010, and 2018 were 0.2802, 0.3867, 0.4729, 0.5471, and 0.5657, respectively. The Δ*f* values were always greater than 0 and exhibited an increasing trend. This indicates that the spatial distribution of cities in the HRB clearly exhibited a diffusion pattern over the past 40 years, with urban development primarily focusing on outward expansion, and this development characteristic has become more pronounced over time.

### 5.3. Structural Correlation Characteristics Between the Water System and Urban System

The fractal structures of the river system and urban system in the HRB are compared from both single-fractal and multifractal perspectives, and the relationship characteristics between the two are analyzed in the following.

#### 5.3.1. Analysis of the Relational Characteristics from the Single-Fractal Perspective

**Figure 9:**
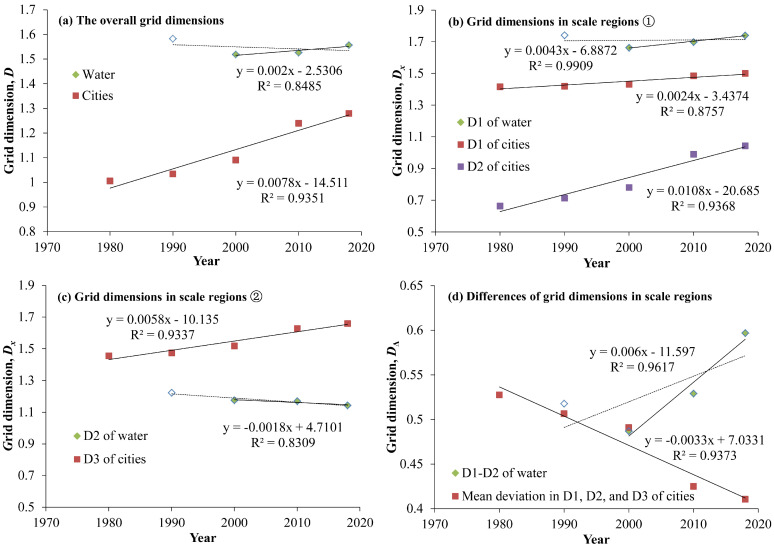
Comparison of the grid dimensions between the water system and urban system in the Huaihe River Basin. The water systems in 1990, 2000, 2010, and 2018 correspond to those in the 1980s, 1990s, 2000s, and 2010s, respectively. (**a**) The overall grid dimensions; (**b**,**c**) the grid dimensions of the sub-scale regions; (**d**) the differences in the grid dimensions among each scale region. Among them, (**b**) includes the grid dimensions of the first-order scale zone of the water system and the first- and second-order scale zones of the urban system; (**c**) includes the grid dimensions of the second-order scale zone of the water system and the third-order scale zone of the urban system. (**d**) The difference in the grid dimensions of the urban system is the average of that across the three scale zones. The lines represent fitted line that varies over time, where the dashed line represents the fitted curve that includes the grid dimensions of the water system in the 1980s, and the solid line does not include that period. The green squares represent water system without 1980s, red and purple squares represent urban system, and white squares represent water system in 1980s.

From the temporal variation in the grid dimension parameters of the urban construction land and water system in the HRB from 1980 to 2019 (Figure 9), the following can be observed: Firstly, under the condition of removing the water system data from the 1980s, except for a slight decrease in the *D*_2_ grid dimension of the water system, the grid dimensions *D*, *D*_1_, *D*_2_, and *D*_3_ of the urban construction land and the grid dimensions *D* and *D*_1_ of the water system all show an increasing trend. Overall and at larger scales, the grid dimensions of the water system were significantly greater than those of the urban construction land, whereas at smaller scales, the grid dimensions of the urban construction land were larger than those of the water system. Some studies suggest that the grid dimensions of the urban areas should not exceed those of the water system; otherwise, this may lead to ineffective utilization of water resources and water shortages in some cities [58]. This result indicates that the spatial filling degrees of the urban construction land and water systems in the HRB generally exhibit an increasing trend; however, at smaller scales, the spatial filling degree of the water system decreased, which may lead to issues of ineffective utilization of water resources and water shortages in some urban areas. Secondly, the *D*_1_-*D*_2_ value of the water system gradually increased over time, while the average difference between *D*_1_, *D*_2_, and *D*_3_ of the urban construction land gradually decreased over time. This indicates that the self-affinity of the urban construction land in the HRB is decreasing, and its fractal structure is optimizing over time; while the self-affinity of the water system is increasing, and its fractal structure is degenerating.

#### 5.3.2. Analysis of the Relational Characteristics from the Multifractal Structure Perspective

**Figure 10:**
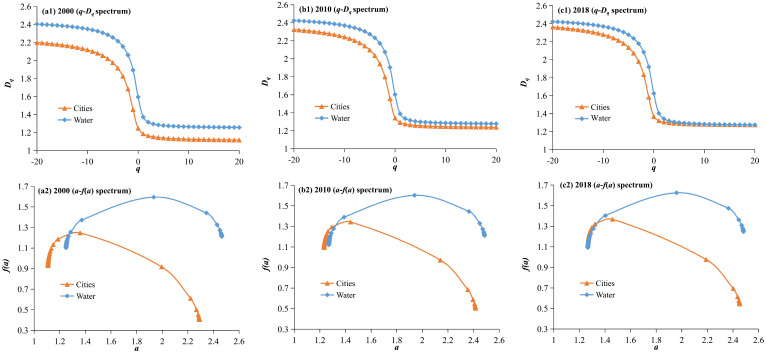
Comparison of the multifractal spectrum between the water system and urban system in the Huaihe River Basin. The water systems in 2000, 2010, and 2018 correspond to those in the 1990s, 2000s, and 2010s, respectively.

To better explore the relationship between the water system and urban system structures, the water system and urban construction land in each period correspond, and the *q*-*D_q_* spectrum and *a*-*f*(*a*) spectrum of them in the three periods of 2000, 2010, and 2018 are plotted (Figure 10). From a multifractal perspective, the correlation characteristics of the water system and urban system structures in the HRB are presented as the follows:

From the perspective of the generalized dimension, *q*-*D_q_*, spectrum, within each *q* value interval, the fractal dimension values of the water system from 2000 to 2018 are all greater than those of the urban construction land, indicating that the development of the water system structure is superior to that of the urban system in the HRB. In terms of temporal variation, although the fractal dimension values of both have shown an increasing trend, the increase in the fractal dimension value of the urban construction land is more pronounced, continuously approaching that of the water system. By 2018, the gap between the two has significantly narrowed, especially in dense areas (q > 0), where the *q*-*D_q_* spectral lines nearly coincide. This indicates that the spatial filling degrees of the urban construction land and water systems in the HRB are continuously increasing, leading to a more complex spatial structure, while the development of the urban structure is more rapid. From the perspective of development trends, the fractal dimension of the urban construction land will exceed that of the water system in the future, especially in urban dense areas or larger cities, which may result in unreasonable utilization of the urban water resources or issues of water source scarcity.

From the perspective of the local dimension, *a*-*f*(*a*), spectrum, the *α*(0) and *f*(*α*(0)) values of the water system during the three periods from 2000 to 2018 were all greater than those of the urban construction land but both exhibited a continuously increasing characteristic. The spatial filling degree of the water system in the HRB is greater than that of the urban construction land, and the trend in the temporal variation is consistent for both, showing a continuous increase in the spatial filling degree and a more complex spatial structure. The width Δ*α* of the singularity index for both the water system and urban construction land also continuously increased, further proving that the spatial filling degree and heterogeneity of both have increased, and the spatial structure has the same development trend. The *a*-*f*(*a*) spectral curve shapes of the water systems and urban construction land differ significantly. The *a*-*f*(*a*) spectral line of the urban construction land shows a distinct unimodal left skew, with a larger range on the right side of the peak, whereas the *a*-*f*(*a*) spectral line of the water systems exhibits a slightly right-skewed unimodal shape, with smaller differences on both sides of the peak. The Δ*f* value of the water system is always less than 0 and shows a decreasing trend. In contrast, the urban system’s Δ*f* value is always greater than 0 and shows an increasing trend. This result indicates that the spatial distribution of the urban construction land is more dispersed, while the water system exhibits stronger aggregation, and this development trend is continuously strengthening. This also reflects that the development of cities in the HRB is primarily characterized by outward expansion, with rapid development of small- and medium-sized towns, leading to an increasing number of the dense urban areas, resulting in an overall dispersed spatial distribution. The development of the water system is primarily based on inherent enhancement; regions with richer water systems experience more rapid increases (or regions with sparser water systems see more significant decreases), leading to an increasingly concentrated spatial distribution.

## 6. Discussions

### 6.1. Urban–Water Relationship

Most cities in China are built near water, which is a common rule for urban site selection [59]. The formation and development of cities rely on a certain scale of water resources, and the quantity and spatial distribution of water systems significantly influence the number, scale, and structure of cities. Research has shown that there is both correlation and similarity between watershed water systems and urban systems, following the same scaling law [49]. The changes in the water system and urban system in the HRB are highly complex and unique, and whether the relationship between the two and their development conform to the general laws of geographical system evolution deserves in-depth analysis and exploration. Based on historical data, the relationship and changes between cities and water systems over a long period can be analyzed, but the results are relatively rough and have low accuracy, making it difficult to depict detailed relational characteristics. The development of remote sensing and geographic information technology has provided new means and methods for studying the relationship between urban areas and water systems. Using remote sensing images can more objectively and vividly display the spatial relationship and dynamic changes between urban areas and water systems. This paper applied fractal theory and GIS spatial methods, based on multi-temporal remote sensing data, to extract information on water systems and urban construction land and analyzed the structural correlation characteristics between the water system and urban system in the HRB. The research results can serve as a reference for water resource management and urban development practices in the HRB, and they offer new insights for deepening the study of watershed urban–water relationships.

To address the conflict between water resources and urban development, a series of policies have been formulated by governments, achieving remarkable results. In 2016, the “River Chief System” and “Lake Chief System” (the principal leaders of Chinese governments at all levels, who act as “river leaders” and are responsible for organizing and leading the management and protection of the corresponding rivers and lakes) began to be implemented throughout the country, which can mitigate the degradation of water systems, and alleviate regional issues of water resources and water environment. Provinces within the HRB have established local standards for water usage quotas, such as Henan Province’s “Water Usage Quota for Industrial and Urban Domestic Purposes”, to address water scarcity during the process of urbanization. The strategy of the “use water resources as its capacity permits” was formulated in 2019, emphasizing the principles of determining urban development, land use, population size, and industrial production based on water availability, ensuring their harmonious coordination with urban development. In the future, it is necessary to scientifically plan urban water systems and attach importance to the protection of lower-grade water systems. Additionally, water resources should be utilized in a conservation-oriented and intensive manner, and the water resources management mechanism should be improved.

### 6.2. Structural Analysis Model

Fractal methods can digitize complex geographical spaces, thereby revealing the spatiotemporal information implied behind geographical elements [50]. They have been widely applied in the study of the structural characteristics and relationships of water systems and urban systems in regions. The application of relevant methods has shifted from single-fractal to multifractal ones, revealing more detailed internal structural characteristics [60]. Research based on fractal theory indicates that the fractal development of urban systems has a certain mathematical relationship with the fractal structures of water systems, where the fractal dimensions of urban systems are less than those of water systems [58]. Research has also demonstrated a symmetrical relationship between water systems and urban systems [49]. The fractal results of regional water systems and urban systems indicate that where there are cities, there must be rivers; however, the presence of rivers does not necessarily lead to the formation of cities. The relationship between the water system and urban system in the HRB is complex, and fractal theory provides a methodological approach to reveal the correlation characteristics of its internal structure. Through fractal research on the water system and urban system in the HRB, we found that although the development of the water system structure is better than that of the urban system, the fractal dimension of the urban system will exceed that of the water system in the future due to the rapid development of cities, potentially resulting in regional water resource shortages. The development direction of the urban system and water system is different; the structure of the urban system optimizes over time, while the spatial structure of the water system continuously degenerates. Additionally, there are differences in their development approaches, as follows: urban development primarily relies on outward diffusion, whereas the development of the water system is mainly based on inherent enhancement. In the future, reasonable water-resource management measures and directions for urban system development should be determined based on the characteristics of the relationship between the water system and urban system structures in the HRB. The structural relationship characteristics between the urban system and water system in different regions of the HRB may vary. Subsequent studies could divide the sub-watersheds to explore the relationship characteristics between the water system and the urban system from a multi-scale spatial unit, providing a scientific basis for more refined water resource management and urban spatial optimization in the HRB.

Compared to the Beijing–Tianjin–Hebei region in northern China [52], both areas display an overall trend of improvement in the water system’s structure at larger scales but face the risk of degradation at smaller scales. The reasons for this difference are that in the Beijing–Tianjin–Hebei region, the South-to-North Water Diversion Project has alleviated water usage issues, while in the HRB, long-term Huaihe River governance projects have contributed to the improvement of its water system. However, both regions should pay attention to the issue of degradation in lower-grade water systems. The fractal structure of the urban system in the HRB exhibits three scale characteristics in contrast to the two scale characteristics of the Beijing–Tianjin–Hebei region, indicating that the overall development of the urban system in the HRB is significantly inferior to that in the Beijing–Tianjin–Hebei area. Because of the influence of human and natural factors, a complete urban system did not form in the HRB over a long period of time. Therefore, in the future, the construction and development of the urban system in this region still need to be emphasized.

### 6.3. Future Improvements

The structural correlation analysis between the water system and urban system in the HRB involves complex human–environment system issues, and the intricate relationships and evolutionary processes between the two systems increase the difficulty of modeling and analysis, resulting in uncertainty in the research results. The main factor affecting the uncertainty of the research results is that different parameter settings in the fractal methods can lead to slight variations in the results, such as the method of grid division and the choice of its size may affect the fractal results. To comprehensively analyze the correlation characteristics between the watershed system and urban system, it is also necessary to include research on the relationship between the urban functional structure and water system structure. In addition, the interpretation of the correlation characteristics between the water system and the urban system in the HRB is limited by the researchers’ professional backgrounds and research objectives, which may introduce certain academic biases. There is a need to explore in depth the connotation of the fractal results and achieve a more scientific analysis and interpretation.

Moreover, remote sensing monitoring of long-term water body changes is an important method for current research on water resource changes [61]. The remote sensing data used in this paper primarily consist of Landsat system satellite images, which can ensure the consistency of the data source. However, because of the differences in image resolution and quality at different times, it may affect the interpretation results of water systems and urban areas. The interpretation method and accuracy also affect the research results. In history, the changes in water systems and urban development were relatively slow, and it is necessary to combine relevant historical data, further revealing the characteristics of the water system evolution and the laws of urban development from a longer time scale. Future research on the evolution of human–water relationships should deepen the understanding of the evolution of watershed water systems and urban development patterns across different temporal and spatial scales. Additionally, modern intelligent analysis and forecasting methods should be introduced to enhance dynamic simulations of the “urban-water” relationship and adaptive strategy research under changing environments, thereby better serving the harmonious human–water relationship in the watershed and the high-quality development of regional economies and society.

## 7. Conclusions

Focusing on the key issues of spatiotemporal evolution of the water system structure and its relationship with the urban system in the HRB, based on long-term remote sensing monitoring data, grid dimension and multifractal spectrum methods were used to characterize the structural evolution of the water system and urban system from different dimensions. Through comparative analysis of fractal parameters and spectral lines, the characteristics and changing patterns of the “urban-water” relationship in the HRB from 1980 to 2019 were revealed. The results are as follows:

(1) Influenced by natural and human activities, the water system structure in the HRB is complex and has obvious scale characteristics, showing optimization at larger scales while continuously degrading at smaller scales. The distribution and development of the water system are primarily characterized by spatial aggregation, and this feature has been continuously strengthening over time.
(2) The spatial filling degree of the water system in the HRB is greater than that of the urban construction land. Both exhibit a consistent trend of temporal variation, showing an increasing spatial filling degree and a more complex spatial structure. However, the self-similarity of the urban construction land has increased, and the spatial structure has optimized over time, while the self-affinity of the water system has strengthened, and its spatial structure has degenerated over time.
(3) Although the development of the water system structure in the HRB is superior to that of the urban system, because of the rapid development of cities, the fractal dimension of the urban construction land will exceed that of the water system in the future, especially in dense urban areas or larger cities, which may lead to unreasonable utilization of the urban water resources or water shortage problems.
(4) The spatial distribution of the urban construction land is more dispersed, with urban development primarily characterized by outward expansion, resulting in an overall dispersed spatial distribution. The spatial aggregation of the water system is stronger, with its development primarily based on inherent enhancement, leading to an increasingly concentrated spatial distribution. This development characteristic and change trend are constantly strengthening over time.

The structure and its evolutionary characteristics of the water system and urban system in this region exhibit both similarities and differences, revealing the issue of water resource scarcity during the process of urban development. In recent years, because of the widespread public attention and the implementation of relevant measures, this situation has improved, exhibiting a positive trend, but the risk of water scarcity still exists. Strengthening water resource management and optimizing the urban system is a long-term and complex process. In the process of urban development, relevant departments need to further strengthen water resource constraints, ensuring that “water determines urban development and social production”, promoting harmony between people and water, as well as regional sustainable development.

Our research highlights the important role of urban morphology and water system structure in assessing the impact of water resources on urban growth, deepening the understanding of the interdependence between urbanization and water availability. This is crucial for regional sustainable urban planning and water resource management. Future research needs to continue exploring and adopting advanced methodologies and incorporating factors such as climate change, socio-economic shifts, and technological innovations to further deepen the understanding of the “urban-water” relationship.

## Figures and Tables

**Table 1:** Grid dimensions of the water system in the Huaihe River Basin from 1980 to 2019.

Period	Grid Dimensions			
	Whole Zone (*D*)	First-Order Scale Zone (*D*_1_)	Second-Order Scale Zone (*D*_2_)	Difference (*D*_1_–*D*_2_)
1980s	1.5829	1.741	1.2232	0.5178
	(0.9946)	(0.9996)	(0.9994)	
1990s	1.5192	1.6622	1.1753	0.4869
	(0.995)	(0.9997)	(0.9994)	
2000s	1.5266	1.6988	1.1696	0.5292
	(0.9933)	(0.9991)	(0.9998)	
2010s	1.5566	1.7396	1.1426	0.597
	(0.9925)	(0.9994)	(0.9996)	

The values in parentheses are the R^2^ from the least squares fitting.

**Table 2:** Multifractal spectrum parameters of the water system in the Huaihe River Basin from 1980 to 2019.

Period	*q*-*D_q_* Spectrum				*a*-*f(a*) Spectrum			
	*D*_0_	*D*_1_	*D*_2_	*D*_1_/*D*_0_	*a*(0)	*f*(*a*(0))	Δ*a*	Δ*f*
1980s	1.6364	1.4070	1.3368	0.8598	1.9564	1.6364	1.2164	−0.2104
1990s	1.5950	1.3719	1.3150	0.8601	1.9333	1.5950	1.2169	−0.1461
2000s	1.6022	1.3890	1.3356	0.8669	1.9390	1.6022	1.2171	−0.1308
2010s	1.6246	1.4033	1.3428	0.8637	1.9588	1.6246	1.2172	−0.1958
Average value	1.6146	1.3928	1.3325	0.8626	1.9469	1.6146	1.2169	−0.1708

**Table 3:** Grid dimensions of the urban system in the Huaihe River Basin from 1980 to 2018.

Year	Grid Dimension				Difference		
	Whole Zone (*D*)	First-Order Scale Zone (*D*_1_)	Second-Order Scale Zone (*D*_2_)	Third-Order Scale Zone (*D*_3_)	*D*_3_–*D*_2_	*D*_1_–*D*_2_	Average Value
1980	1.0059	1.4155	0.6639	1.4553	0.7914	0.7516	0.7715
	(0.9817)	(0.9998)	(0.9922)	(0.9983)			
1990	1.0337	1.4194	0.7138	1.4739	0.7601	0.7056	0.7329
	(0.9837)	(0.9999)	(0.9943)	(0.9983)			
2000	1.0905	1.4306	0.7812	1.5178	0.7366	0.6494	0.6930
	(0.9866)	(0.9999)	(0.9949)	(0.9986)			
2010	1.2391	1.486	0.9904	1.6281	0.6377	0.4956	0.5667
	(0.9915)	(0.9998)	(0.9964)	(0.9993)			
2018	1.2792	1.5	1.0429	1.6591	0.6162	0.4571	0.5367
	(0.9926)	(0.9996)	(0.9971)	(0.9995)			

Note: The values in parentheses are the R^2^ from the least squares fitting.

**Table 4:** Multifractal spectrum parameters of the urban system in the Huaihe River Basin from 1980 to 2018.

Year	*q*-*D_q_* Spectrum				*a*-*f(a*) Spectrum			
	*D*_0_	*D*_1_	*D*_2_	*D*_1_/*D*_0_	*a*(0)	*f*(*a*(0))	Δ*a*	Δ*f*
1980	1.1962	1.1292	1.1033	0.9440	1.3178	1.1962	1.1842	0.2802
1990	1.2120	1.1472	1.1225	0.9465	1.3291	1.2120	1.1842	0.3867
2000	1.2475	1.1865	1.1641	0.9511	1.3590	1.2475	1.1847	0.4729
2010	1.3424	1.2927	1.2753	0.9629	1.4394	1.3424	1.1847	0.5471
2018	1.3676	1.3219	1.3066	0.9666	1.4587	1.3676	1.1848	0.5657
Average value	1.2731	1.2155	1.1944	0.9542	1.3808	1.2731	1.1845	0.4505

## Data Availability

The source of the data used in this research is provided in Section 4.
